# Equivocal Justice: Migrant and Refugee Survivors of Family Violence and Interactions With the Australian Legal System

**DOI:** 10.1177/10778012251334767

**Published:** 2025-04-21

**Authors:** Claire Sullivan, Karen Block, Jeanine Hourani, Cathy Vaughan

**Affiliations:** University of Melbourne, Melbourne, VIC, Australia

**Keywords:** migrant and refugee women, family violence, domestic violence, legal system, legal consciousness

## Abstract

Little is known about how migrant and refugee women who experience family violence interact with the Australian justice system. Drawing on interviews with survivors and service providers and a focus group discussion (*n* = 73), this paper explores how survivors view and engage with Australian legal interventions. We found the legal system to be an unreliable site for migrant and refugee women; it was capable of both perpetuating violence through institutional discrimination and offering much-needed protection, at times simultaneously. Interactions were influenced by and influenced survivors’ legal consciousness and informed their relationship with the resettlement state. The findings underscore the importance of legal responses premised upon listening to survivors’ claims, respecting their agency, and accounting for the intersectional realities of their lives.

## Introduction

Many migrant and refugee women in Australia must at times engage with the justice system following family violence, by choice or otherwise. How the system responds is of vital consequence. International and Australian research confirms that migrant and refugee women who have experienced family violence face multiple systemic barriers to accessing legal institutions (Burman & Chantler, 2005; Gillis et al., 2006; Guruge & Humphreys, 2009; Raj & Silverman, 2002). These include limited legal knowledge, fear of authorities, poor police responses, racism and discrimination, language barriers, and inadequate interpreter services (Ghafournia & Easteal, 2021; O’Neal & Beckman, 2017; Vaughan et al., 2016; Zannettino, 2012). Additional structural challenges such as housing insecurity, financial hardship, and physical and social isolation further complicate help-seeking efforts (Block et al., 2022; Hourani et al., 2022; Murray et al., 2019). Although these barriers are now relatively well-documented, less is known about how migrant and refugee women engage with and experience the legal system (Block et al., 2022; Lemma et al., 2023; Sullivan et al., 2023). This study aims to address this gap, exploring migrant and refugee survivors’ views of and interactions with the justice system in the State of Victoria, Australia.

The paper is structured as follows: we first introduce how we position and understand the legal system, framed through an intersectional, sociolegal lens, and detail the relevance of the concept of legal consciousness. We then briefly discuss definitions of family violence in relation to migrant and refugee communities, followed by an outline of the limited existing evidence surrounding migrant and refugee survivors’ experiences of the legal system, highlighting the need for this research. Our qualitative research methods are then detailed, describing the collection of interviews with migrant and refugee survivors (*n* = 24) and service providers (*n* = 42), as well as a small focus group discussion (FGD). We next describe our findings about migrant and refugees’ ideas about and interactions with the legal system and illustrate the risky nature of engaging with a system embedded in systemic inequities. Fears of authorities and failures of authorities are elaborated, as well as survivors’ difficulties navigating multiple jurisdictions with differing priorities, in particular family and migration law regimes that fail to hear and protect survivors of violence. Notwithstanding this, we highlight contrasting narratives of empowerment found in the data and how such narratives can coexist with experiences of structural and interpersonal discrimination, revealing a potential dissonance experienced by survivors seeking legal remedies to violence. The final section explores the impact of survivors’ experiences and perceptions of the legal system on personal and collective legal consciousness and conceptions of belonging or alienation within Australia. We touch upon potential reforms in police and court approaches to enhance procedural fairness for migrant and refugee women, noting, however, that survivors’ empowerment through the legal system is not possible without sufficient social, material, and housing resources, regardless of relationship and immigration status, to provide migrant and refugee survivors of violence with the necessary security to ensure space for agency and safety.

### The Legal System and Legal Consciousness

The “justice/legal system” in this paper is understood in a sociolegal sense to encompass the role of law in everyday life or law-in-action, focusing on interactions with legal actors, institutions, and frameworks. Indeed, we view the legal system as a social site involving many spaces and actors, such as law enforcement agencies and agents; courts, tribunals, judiciary and court personnel; legal practitioners; and social support workers (Yngvesson, 1988). Legal responses to family violence considered in this study entail many legal jurisdictions spread across a range of courts, including civil protection law, criminal law, child protection law, family law, and immigration law. The various spheres of the justice system do not operate in isolation for migrant and refugee women, though they do jurisdictionally and doctrinally. Our recognition of the breadth of the legal system as experienced by women is in keeping with an intersectional feminist approach that emphasizes relationality and the social context of experiences, rather than viewing experiences in superficial isolation as is traditional in legal approaches (Collins, 2019; Minow, 1990).

An orientation toward law-in-action as it affects migrant and refugee women also underpins the concept of “legal consciousness,” which describes the way individuals understand and experience the law and its significance to themselves (Merry, 1990, 2012; Silbey, 2005). Legal consciousness amounts to more than one's knowledge or ignorance of the law; rather, it denotes the way in which law influences one's understanding of their social world, shaping cognition and actions (Chua & Engel, 2019). It is informed by and informs individuals’ beliefs about their rights to access the law and is mutable and contingent (Abrego, 2011; Merry, 2012). The legal system, and thus legal consciousness, are experienced differently depending on an individual's social location within intersecting hierarchies of power associated with race, gender, class, sexuality, and other positionings (Crenshaw, 1989; Sokoloff & Dupont, 2005).

In recent years various studies have begun to explore the legal consciousness of migrant groups (Abrego, 2011; Graca, 2018; Güdük & Desmet, 2022; Miežanskienė, 2020). Migration to a new legal jurisdiction can disrupt and reshape legal consciousness (Güdük & Desmet, 2022), with such being influenced by interactions with the law following family violence, as explored in this paper.

### Definitions of Family Violence and Migrant and Refugee Populations

In this paper, we refer to the broad legal definition of family violence in Victoria, namely any behavior by a family member toward another family member that is physically, sexually, emotionally, psychologically, or economically abusive, threatening, coercive or controlling (*Family Violence Protection Act 2008* (Vic), s5). The violence that migrant and refugee women experience is intersectional; it is shaped by women's particular social location, influenced by race, gender, and class, as well as historical factors, such as experiences of conflict and migration (Sokoloff & Dupont, 2005). Research suggests that in addition to forms of abuse experienced by all populations, migrant and refugee women may be vulnerable to particular forms of violence such as immigration-related abuse (whereby perpetrators coercively control survivors through the use of visa status), reproductive coercion, and multiperpetrator violence, which are caused by or exacerbated by structural violence (El-Murr, 2018; Suha et al., 2022). For example, state policies that foster survivors’ precarity through unstable visa status and limited access to services/welfare are forms of structural violence further exploited by perpetrators (Hourani et al., 2022; Maher & Segrave, 2018; Vasil, 2024). There exists a danger when discussing family violence and migrant and refugee populations that particular experiences of violence may be racialized or framed in terms of “culture,” obscuring underlying structural factors (Burman et al., 2004), which was a tension that also arose in our data (discussed further below). Literature also indicates that the broad legal definition of violence applicable in Victoria may be rejected by some migrant and refugee survivors in favor of their own definitions, focusing in particular on physical violence (Lemma et al., 2023; Satyen et al., 2018).

### Migrant and Refugee Survivors, Help-Seeking, and the Legal System

Women of all populations are at risk of experiencing further victimization when dealing with law enforcers and court systems due to patriarchal norms and discrimination (Campbell & Raja, 2005; Erez & Belknap, 1998; Gillis et al., 2006; Moore, 2019). Evidence suggests that police responses are critical in shaping women's experiences of the legal system and their adequacy likely predicts whether a woman will call the police again (Fleury-Steiner et al., 2006; Goodman-Delahunty & Crehan, 2016). A study into police responses in Queensland, Australia, not specifically concerned with migrant and refugee women, reported various concerning responses by police, such as a failure to investigate claims or take appropriate action, minimization of violence, victim-blaming, and aligning with the perpetrator (Douglas, 2019). Some literature indicates that perceived procedural fairness within the legal system may matter more to survivors than the actual outcomes (Bell et al., 2011; Goodman-Delahunty & Crehan, 2016).

Migrant and refugee survivors are likely to also face unique forms of systemic violence within and outside the legal system (Hourani et al., 2022; Raj & Silverman, 2002). Experiences of institutional racism when help-seeking can be particularly distressing for survivors of violence (Ghafournia & Easteal, 2021; Losoncz, 2019). Concern about racist treatment by authorities not only relates to survivors themselves, but also perpetrators, particularly in the context of antimigrant public discourse; legal intervention may risk exposing “a family to public shame and invites the intrusion of harmful federal immigration and protective services in the lives of victims. Instead of being helped, victims may often find themselves under surveillance or persecution” (Erez et al., 2009; Kulwicki et al., 2010, p. 732).

Research has found that migrant and refugee women are invisible in much family violence policy in Australia (Ghafournia, 2011; Ghafournia & Easteal, 2018). One study indicated that an intersectional approach was not embedded in most family violence policies and services, which fail to acknowledge the role of racism and white privilege in the experiences of refugee and migrant women (Maturi & Munro, 2023). The authors found that notions of “culture” are mobilized to essentialize and racialize migrant and refugee communities experiencing family violence (also see Adelman et al., 2003; Burman et al., 2004).

Limited research is available specifically exploring migrants’ and refugees’ experiences with police and within courts following family violence in Australia and elsewhere. A Royal Commission into Family Violence in Victoria identified systemic police failures in their dealings with migrant and refugee survivors, including instances of culturally insensitive police responses and serious interpreter issues such as the failure to use an interpreter, the use of a family member, poor quality interpreters, the use of interpreters known to the perpetrator/survivor, or the even use of the perpetrator themselves (Neave et al., 2016). Concerns have been raised about the ability of migrant and refugee survivors to communicate their version of events, with migrant and refugee women regularly misidentified by police as primary aggressors (Ulbrick & Jago, 2018). Migrant and refugee survivors may be apprehensive about involvement with authorities due to fear of being separated from their children by child protection authorities (Maher & Segrave, 2018; Robinson et al., 2025). It should also be noted that many migrant and refugee survivors come into contact with authorities without contacting the police themselves (Barrett et al., 2011); in a US study with 137 migrant survivors, a third of those involved with the criminal justice system did not make initial contact with the police (Erez et al., 2009).

Evidence indicates that court environments present challenges with inadequate multilingual resources/orders (Neave et al., 2016) and inconsistent interpreter availability, including a lack of available female interpreters (Neave et al., 2016; Sullivan et al., 2023; Troshynski et al., 2021). In a study exploring migrant and refugee women's experiences of violence and help-seeking in Australia, Vaughan et al. (2016) found that perpetrators mobilized the court system to harass and intimidate migrant and refugee survivors by making crossclaims which were difficult for survivors to defend, or by issuing proceedings in the family court, compromising the well-being of both the women and their children.

Crucially, many migrant and refugee women fear and/or experience immigration status repercussions for themselves, their children, or the perpetrator when they seek help from legal authorities following family violence (Anitha, 2011; Burman & Chantler, 2005; Vasil, 2024). Pathways exist for women on partner visas to gain permanent residency if they have experienced family violence; however, such claims are difficult to navigate and evidence (Borges Jelinic, 2019; Segrave, 2017). Borges Jelinic (2021) found that such women are subject to protracted processing times, cultural insensitivity, and procedural inconsistencies when dealing with the immigration department, ultimately undermining the law's purported purpose of protecting those on partner visas. Pathways to residency for victims on other types of temporary visas, such as asylum seekers, students, or most workers, are less clear and even more difficult to navigate. Survivors must also contend with the possibility that their partner or they themselves may have their visa canceled if they are found to have committed family violence^1^; a real risk given how commonly migrant and refugee women are misidentified as primary aggressors and the use of crossclaims by perpetrators (Ulbrick & Jago, 2018; Vaughan et al., 2016).

## Methods

This paper draws upon two qualitative data sets collected by the authors exploring migrants’ and refugees’ experiences of help-seeking following family violence in Australia. The first comprised part of the SEREDA project (*SExual and gender-based violence against Refugees: Experiences from Displacement to Arrival*), a wider multicountry study that took place in England, Turkey, Sweden, and Australia (University of Birmingham, 2023). It involved interviews with Australian service providers and survivors, conducted between 2019 and 2020, exploring refugees’ experiences of sexual and gender-based violence including family violence, and the responses and support provided. The third author, fluent in Arabic and English, undertook these interviews. The second data set consisted of interviews and a FGD conducted between 2020 and 2024, focusing specifically on migrant and refugee women's views of and experiences with the legal system following family violence in Australia. The first author conducted these interviews and FGD for the second data set. Interpreters were used where required.

A total of 73 participants took part in the respective data collections. The first data set consisted of interviews with 38 participants (*n* = 22 service providers; *n* = 16 women from refugee backgrounds). The second data set consisted of interviews with 28 participants (*n* = 20 service providers; *n* = 8 migrant and refugee women) and one FGD with service providers in regional Victoria (*n* = 8). One person participated in an interview and the FGD.

The service providers represented in both data sets included a range of professionals working in migrant/refugee services or sexual and gender-based violence/family violence response services, including community-based and police lawyers; court support personnel and volunteers; caseworkers and advocates; multilingual health educators; community engagement, violence prevention and policy practitioners; and community advocates and counsellors. The authors purposively recruited service providers through their networks to include a breadth of roles.

Migrant and refugee women were primarily recruited with the assistance of community-based organizations that work with survivors of family violence based in Victoria. Women who participated in the earlier interviews were originally from Syria (6), Iraq (5), Lebanon (3), Egypt (1), and Eritrea (1). Women who participated in the later interviews were from China (1), South Sudan (1), Somalia (2), Kenya (1), Cambodia (1), and India (2). Although the SEREDA study was nominally concerned with “refugees,” the term refugee was defined broadly to encompass temporary migrants who were not legally considered refugees but found refuge in Australia via other migration channels, such as spousal visas or student visas. A distinction between migrant and refugee was not made during collection of the second data set, as the delineation between categories was not deemed clear or useful for the objectives of the analysis. Indeed, many women, including women in this study, move from the category of “temporary migrant” to “legally recognized refugee” during their migration journey, or never receive official refugee status despite meeting the legal criteria. We also identified that many of the issues raised by participants in the first study were likely to also be experienced by migrants regardless of their immigration status.

Interviews with service providers ranged from 40 to 120 min, generally via teleconference. Interviews with migrant and refugee women ranged from 30 to 160 min and where possible were conducted in person to better ensure participant safety and support. Some interviews were broken into two sessions when deemed appropriate by either the participant or researcher.

The projects received ethical clearance from the University of Melbourne Human Ethics Sub-Committee (ethics ID numbers: 1954369.1 and 1955379.1). A safety protocol based upon trauma-informed principles (Vaughan et al., 2016) was utilized for all interviews to support and protect participants and researchers.

Interviews were audio-recorded and transcribed with participant consent, with notes used instead for three participants who declined recording. Pseudonyms were given to all survivors to protect anonymity. Service providers are denoted by their professional role. The first author analyzed both data sets contemporaneously to investigate participants’ perceptions and experiences of the legal system. Data were analyzed thematically according to the steps outlined by Braun and Clarke (2006) using NVivo as a data management tool.

## Findings

The findings indicate that migrant and refugee women may fear and avoid interacting with legal authorities following family violence, with many women viewing formal law and authorities as either hostile or irrelevant to them. Such positionings in relation to the law were sometimes due to a lack of legal knowledge, although apprehensions were also well-founded as forms of institutional discrimination did characterize many women's experiences with the police and courts. Indeed, women who wanted to stay in relationships were potentially subject to systemic alienation, with inadequate legal or support options available. The data also indicated that existing legal frameworks failed to effectively capture the specific types of violence experienced by migrant and refugee women or that these frameworks were inappropriate or politicized. Participants struggled with inconsistent approaches to family violence issues across different jurisdictions. Notwithstanding this, narratives of empowerment also featured in the data, at times alongside experiences of alienation.

### Knowledge and Fear of the System

Many participants reported apprehension about calling authorities to assist with family violence, sometimes conveying a positioning “against the law” (Ewick & Silbey, 1998). One narrative observed in migrant and refugee survivors’ and service providers’ interviews was that any involvement of authorities would inevitably lead to separation of the family unit or loss of custody of children (Maher & Segrave, 2018; Robinson et al., 2025). We found perpetrators could manipulate such notions to threaten women against making reports of violence to authorities.

Fear of calling the authorities could also be due to apprehension about community judgment (Graca, 2018). Both migrant and refugee survivors and service providers noted that migrant and refugee women may face social and cultural taboos concerning police and legal involvement. One participant explained why she avoided seeking police help even though her partner was threatening her life with a knife:

I should have called [the police] but I didn't…. Back home when we left you see, they said to us, ‘Now you need to go to Australia and you need to be a good family, you need to stay together and support each other.’ You can't call the police. (Jasmin)

Jasmin's story is exemplary of views that involvement of authorities would undermine the integrity of the family unit. Our findings also indicate that fear of engaging with the law could be due to poor experiences with legal authorities in countries of origin or transit and a lack of understanding of how the legal system may differ in Australia (Vaughan et al., 2016; Zannettino, 2012). This highlights the potential complexity of legal consciousness experienced by migrants and refugees, who are required to integrate their multiple relationships to various states’ legal frameworks and their transnational social location (Kubal, 2013; Kulk & De Hart, 2013). It was reported that perpetrators also regularly used a greater command of English to misrepresent the Australian legal system and manipulate women's understanding of their position before the law:

I had no idea that I was able to get a divorce here – I didn’t know. The only thing he used to tell me about Australia was that if you misbehaved in public, they take your kids away from you and put you in prison. (Sakina)

Some survivors also conveyed that negative experiences with Australian government agencies, particularly police, could lead to mistrust of external authorities in general: “Yeh, I didn’t know where to go. You know, the police here, they give you bad advice” (Rasha). In quotations such as this, participants positioned themselves as alienated from the legal system, viewing the legal system as either irrelevant or actively antagonistic toward them and sought to evade legal intervention (Halliday, 2019; Hertogh, 2018). One service provider working with a newly arrived community observed: “They don't trust the government. They don't trust the legal system. So how can they trust that something good will happen for them?” (Caseworker 2).

Service providers in particular noted that migrant and refugee women often have limited understanding of how family violence is legally defined in Australia, reporting that some do not consider sexual abuse (including marital rape), emotional abuse, financial abuse, or controlling behaviors to comprise family violence deserving of legal recourse. Unsurprisingly, lack of knowledge around what constitutes family violence legally made engaging with the Australian legal system more difficult, particularly where women did not have legal representation or support to navigate legal forms and understand specialized legal language. Dissonance between survivors’ own definition of violence and legal definitions could also lead survivors to avoid or disengage with legal interventions (Lemma et al., 2023).

Service providers consistently highlighted the lack of appropriate legal education available for migrant and refugee women. For women who arrive on refugee visas, limited legal education sessions are provided; however, service providers lamented that nothing comparable existed for women arriving on other visas, such as spousal visas, meaning that although legal immigration avenues exist to help women experiencing violence in these situations, women were not aware of them. It should be noted that the sessions delivered to refugee entrants were also critiqued; one service provider who had helped deliver the program for a group of women who had no or low literacy skills noted: “It's just let's push out all this information and hopefully some of it sticks so we can tick the box, and claim the money for saying that we've educated them about the legal system in Australia” (Caseworker 1).

Indeed, service providers emphasized the need for culturally appropriate legal education that is meaningful to different migrant and refugee communities. Several described the importance of women's groups and community organizations, stressing the need to build trust. One bilingual health educator felt that legal education was most effectively delivered through one-on-one casual discussions, such as regular coffee dates or during other social activities. This observation emphasizes the relationality of legal consciousness and how understandings and responses to the law evolve through relationships with others (Abrego, 2019).

### Legal Recognition of Different Forms of Violence Faced by Migrant and Refugee Women

Our findings indicate that family violence legal frameworks and responses may not appropriately or adequately account for particular types of violence that many migrant and refugee women face. These included multiperpetrator violence, immigration-related violence, forced/early marriage, and marriage/divorce-related violence. Such experiences could position women outside of the ambit of the law or complicate the claims they were able to make in the legal system, potentially resulting in alienation.

Participants frequently raised the complexities faced when there were multiple perpetrators of violence, who may or may not include their partner. At times, violence from other family members (particularly families-in-law) amounted to domestic servitude, which was not acknowledged through their experiences of the legal system (notwithstanding federal modern slavery legislation which could be applicable).

We found that multiperpetrator violence intersected regularly with immigration-related violence, another frequently reported form of violence not well accounted for by the various legal spheres which respond to violence. Immigration-related violence is not a crime in Victoria but could be considered a form of coercive control under family violence protection provisions. Concern was raised by some service providers about the immigration department's failure to take a consistent family violence lens that protects survivors. One participant described a case where a survivor's partner made false claims to hinder her visa application, which were accepted by the department despite substantial evidence of a history of violence: “I just couldn't believe that they would use that evidence against her and not question it at all” (Legal practitioner 3). Perpetrators’ immigration-related threats concerned women and their children's status but also their own: “The migration space is very much used as a tool of family violence by perpetrators… There's the guilt of: ‘You're going to get my visa cancelled’” (Legal practitioner 2).

The vulnerability of those on temporary visas to immigration-related violence has been highlighted in previous research (Segrave, 2017; Vaughan et al., 2016). We found that women on permanent visas could also be subject to immigration-related violence, as even permanent visas can be canceled. For some, perpetrators’ threats were false and powerful due to women's lack of knowledge of the legal system (Erez et al., 2009). Conversely, threats also arose out of legal awareness, as a court order related to family violence might lead to visa cancellation. This highlights the complex legal awareness needed by migrant and refugee survivors.

Another form of family violence experienced by migrant and refugee women that arose in the data was early/forced marriage. One service provider criticized the fact that support for women seeking to escape a forced marriage depended on their participation in the federal criminal justice process:

[T]here's the possibility that [their] family could be investigated and prosecuted… most clients… don’t want to lose their family but they just don’t want to get married. It doesn’t matter their age or what community they’re coming from. (Casework manager 2)

Some of the migrant and refugee women (*n* = 6/24) interviewed had experienced forced/early marriages in their country of origin, but none had engaged with the legal system about these issues. Often, forced or early marriages intersected with other forms of family violence, though not all women saw such marriages as inherently violent. One woman we interviewed was married at 14 to a man 12 years her senior and did not view this as problematic. A police prosecutor in civil protection proceedings noted that some colleagues saw early marriage as irrelevant to current safety concerns, while she viewed it as contextual information for understanding violence dynamics: “I took [early marriage] to be something quite significant whereas other people were not looking at that… There's got to be a whole history there if she was married at 14” (Legal practitioner 1). The family violence protection jurisdiction is concerned with current and future safety risk rather than past risk, but this quotation reveals the complexity associated with assessing present and future risk, particularly in relation to migration or cultural factors that may be poorly understood by legal actors. Literature confirms that complex considerations of past, present, and future risk are also relevant in the context of asylum law and family violence; however, decision-makers tend to inappropriately focus on future risk only (Aitchison, 2024).

Several participants also raised concerns that the legal system paid little attention to migrant and refugee women's other experiences of marriage and divorce-related violence. Such violence took different forms, generally operating at the nexus of religious and legal marriage (Ajlan, 2022), including divorce refusal, experiences or threats of polygamy, false proposals, and fake religious marriages. In divorce refusal cases, perpetrators withheld a religious divorce while seeking a legal divorce, leaving them free to remarry while the survivor was left in limbo, remaining religiously married:

I had been asking him to get a divorce for seven years, but he wouldn’t divorce me, he would say ‘I’ll marry 10 other women as other wives and I’ll continue to be violent towards you until you die’ and he would say all this stuff in front of my kids. (Aliyah)

Abuse surrounding marriage registration was also raised. One participant described how her partner deceived her by never registering their marriage: “He said to the Sheikh, ‘Let me get married now and then, after a while, I'll legally divorce the other one and register this marriage’ but then he never did that” (Alma). One service provider noted a tendency for perpetrators to withhold access to marriage documentation obtained overseas, impeding women's immigration claims. These forms of marriage and divorce-related abuse could complicate and potentially deprive women of legal mechanisms of redress following family violence.

When discussing the particular types of violence that migrant and refugee women face, several participants highlighted the danger of stereotyping or racializing communities. One participant noted that this was particularly problematic where legal narratives reinforced ideas about Muslim communities that promote Islamophobia, and described how forced marriage laws had been politically mobilized:

It was something that Government could really sell, and everybody wanted a piece of those young women and those stories… [H]ow do we do this work, and [work] with [Australian Federal Police]?… it's felt, and it continues to feel at times, very uncomfortable as to how to ethically manage that tightrope. (Casework manager 1)

This quotation reveals a tension between recognizing unique experiences of violence, and inadvertently contributing to racialization and discrimination against migrant and refugee groups through legal doctrine and interactions. Additionally, migrant and refugee women's particular experiences of violence, as examined here, should not be understood simply as “cultural” forms of violence, obscuring structural and intersectional underpinnings (Maturi & Munro, 2023).

### Legal Support to Stay in Relationships

Our findings indicate that the legal system is at times a difficult space to inhabit for women who want to remain in a relationship deemed abusive. Various service providers suggested that the legal system was tilted toward assisting women to leave, rather than assisting them to stay safely in relationships. We found that migrant and refugee women—like all women—had to make their own safety analysis, weighing up the risk that legal interventions may escalate violence. Indeed, research confirms that leaving an abusive relationship is likely dangerous for many women (Spearman et al., 2023). Both survivors and service providers noted that safety consequences may be greater if legal interventions are subject to social taboos or there is pressure from communities to remain in relationships.

Our research indicated that migrant and refugee women who sought to maintain their relationships were likely to seek to avoid criminal charges being laid against their partners. However, if police have enough evidence, they may proceed with criminal charges without the support of the survivor as a witness, pursuant to a proarrest and procharge policy (Victoria Police, 2022). As a result, women could find themselves enmeshed in multiple, complex legal interventions in which they experienced a lack of agency. Some service providers reported that such stories act as a deterrent to others from accessing the legal system. Several participants reported strategies used to try to resist the legal consequences of interacting with authorities, such as withdrawing their witness statements in civil protection or criminal proceedings. It should also be noted that some migrant and refugee women who *did *want to separate from their partners wanted to protect their abusers from criminal punishment for various reasons, such as to avoid a criminal record (Lemma et al., 2023).

### Failure of Police Responses

Several migrant and refugee women we interviewed reported being ignored or disbelieved by police, a concern echoed by service providers. Factors such as communication barriers, limited physical evidence of violence, and perpetrators contacting the police first to control the narrative, all contributed to silencing survivors: “And it's been like the police showed up, he had scratches on him that came from self-defence. There's no interpreter and then a protection order has been made against her” (Legal practitioner 2). One participant, Rita, called police after her partner threatened to kill her in front of her son. Without an interpreter present, the police failed to ascertain what happened, and left without taking any action:

And [the perpetrator] started to laugh and said to me ‘Those? Those are the police that you called to do something to protect you? That's who you called? What did they do? They didn’t do anything! They were laughing and joking with me and said bye and just left. (Rita)

This resulted in an escalation of violence and during a later incident Rita called the police again. On this occasion there was clear physical evidence on her face of being beaten so the matter was treated seriously by police and a protection order was made. Despite this, Rita has since had difficulties asking the police to take breaches of the protection order seriously. Service providers also noted that the failure of police to respond to breaches of orders was consistently frustrating (Dowling et al., 2018).

Poor experiences with police affected attitudes toward police and authorities generally and the likelihood of survivors contacting authorities again. Nala described a history of severe family violence, including physical beatings and threats to kill. The police made an application for a protection order but she convinced the police and court to drop the application as she wished to maintain her relationship. When she finally separated from the perpetrator, she sought protection from police due to her ongoing fears. The police chose not to pursue a protection order because the perpetrator was not presently harassing her, seemingly without regard to their history or the dangers associated with separation:

They said to me, ‘As long as he's not calling you or he's not coming at you. That's fine. You don't need to get [an order]….’ I wouldn't [call the police again] because I understand it's a waste of time.

Nala's former partner would go on to make a false report to child services and police, causing significant distress, and further loss of trust in the legal system.

Service providers confirmed that those who were misidentified as primary aggressors were particularly likely to mistrust the legal system. One legal practitioner described how difficult it was to correct the record when a victim is misidentified:

We are seeing more of those mis-ID cases popping up and it does generally seem to be women who are from – perhaps they're new arrivals or they're from a non-English speaking background and often… they didn’t call the police first. So his story is believed first… I have had some cases where I've been able to change that narrative. It's not always easy and it involves a lot more work and you're liaising with the police… But it really puts those women under even more pressure. (Legal practitioner 4)

Such situations underscore the importance of legal representation for migrant and refugee women; however, it was noted that many women did not have access to adequate representation.

Although many instances of police failure and discrimination were reported, participants also emphasized that there have been improvements in police approaches to migrant and refugee women in recent years. Service providers spoke particularly highly of specialized family violence police units. However, this also highlighted inconsistency in police responses: “it all depends whom you get on the day, really” (regional FGD participant). This was particularly so in regional areas. One legal practitioner working in a regional city cited police stations that she told clients to avoid when reporting family violence issues such as breaches. Service providers in the regional FGD observed that problems arose in the circumstance where the police officers knew the perpetrator, dismissing the allegations of the survivor.

### Court Interactions

Participants reported that navigating the civil protection and criminal regimes was often a disorienting experience for migrant and refugee women:

Especially [women] from a different cultural background—if they are really not educated, recently arrived, first time reported to the police, first time in court and the system—they just don't understand what's happening. We don't expect them to understand everything…. They will just know they have got [their order] and that's what they need to follow… The papers that they can't read. And court language is quite difficult to understand. And even if they say, “Yes, I'm understanding,” when they get out of the courtroom, they're just lost. (Court support worker)

Several other participants also highlighted the problems associated with providing women with orders that are in English. This relied on Magistrates, court personnel, or lawyers explaining the order through an interpreter, which often did not occur. Such problems extended to additional support services which the court may refer survivors and perpetrators. One participant described responding to Magistrates’ requests to provide lists of services:

I'm like, ‘Yeah, sure, no worries,’ and I'm highlighting [the list], and I'm just thinking, ‘This is in English… if they call up this organisation, does this organisation have the ability or the capability to even handle that matter?’. (Legal practitioner 1)

Issues with court interpreters and communication challenges were particularly problematic for migrant and refugee women (Sullivan et al., 2023). Often male perpetrators had better English than their partners due to access to education or being Australian-born. Service providers noted that interpreters were not available in some languages, requiring people to use their second or third language. Moreover, in-person interpreters were difficult to obtain regionally, leading to a reliance on telephone interpreting. Migrant and refugee survivors and service providers expressed concern about inadequate interpreter services: “It is just fraught with possibilities of misrepresentation, and disempowerment. Very concerning at times that people aren't getting a fair go” (Court support volunteer 2).

Time pressures were highlighted as another problem. Court lists were often overburdened, and duty lawyers were unable to advise all those in need, leaving many unrepresented or underrepresented. One service provider described a case where a survivor was not informed that her protection order matter was listed in the criminal court not the civil court upstairs. The hearing proceeded without her, the order was not explained, and a mistake in the order required another court visit. The participant reflected: “they feel like they're traumatised again because of the system. Sometimes, they're really good… And sometimes, [there's] no one to take care of it” (Court support worker). This participant noted that such issues affect Australian-born women as well migrant and refugee women.

However, working with interpreters meant that matters tended to take much longer. Participants raised frustrations that, despite this, interpreters were typically booked by in the Magistrates’ Court for only half the day, exacerbating the pressure for migrant and refugee women's matters to be resolved quickly: “The translators will only be available until lunchtime, and that means that everything has to get on before then… I often feel at the end of it that the person hasn't really understood what has happened” (Legal practitioner 5).

One participant, Fatima, noted that although she spoke some English, her English was affected by her emotional state and stress in the courtroom environment, so she would have benefited from an interpreter. Indeed, Fatima was not offered a lawyer, a support person, an interpreter or any help by court personnel at court when she presented at a Magistrates’ Court to make an application for protection. She found filling in the application forms and representing herself in the courtroom before the Magistrate challenging: “things would be different if people had just asked me ‘Do I need a support person’ [and] ‘Do I need an interpreter’” (Fatima). Nor did the court explain how her husband would be served with the interim order and Fatima was shocked when the police arrived at their house where she was living with the perpetrator. She asked the officers if there would be criminal charges, but they refused to speak about the matter. The court also recommended that her husband complete a men's behavior change program, but, as a temporary resident, he was ineligible for a funded program. Although she was pleased with the order that was the legal outcome, Fatima viewed her experiences—and later interactions with the police—as discriminatory denials of procedural justice:

I feel the justice system actually doesn't see me or hear me as a migrant, you know. Like you know, I really question sometimes if it would be the same response from the police or the legal system if this would happen to someone who is native here.

Fatima's simultaneous satisfaction with the legal outcome and serious concerns about the process highlight the complex and equivocal relationship some women had with the justice system, which could involve both mechanisms of protection and discrimination. For others, experiences of the legal system resulted in an overall sense of disempowerment:

There's no justice. People can cheat the system easily. The law doesn’t do anything. It exists but it's failing…. My mental health is now worse than it was before because of the lack of action by services around family violence. (Mariyan)

Two service providers themselves described the Magistrates’ Court as a hostile environment to work in, with an exclusionary culture:

The court personnel treat me badly and I work there. They are just dismissive and don't give you the time of day. It must be really difficult for migrant women or vulnerable groups to communicate and get help if I find it hard. (Court support volunteer)

It was suggested that this dynamic could partly reflect a lack of diversity in court personnel, including legal practitioners and the judiciary: “I think most of us are still predominantly white” (Legal practitioner 1).

### Enmeshment in Multiple Jurisdictions

Concerns were raised in relation to the contradictory ways different jurisdictions treated family violence experienced by migrant and refugee women. Migrant and refugee women could find themselves in multiple jurisdictions alongside the civil protection jurisdiction, often requiring different legal practitioners. One survivor noted that attending different courts had become like a job as it required so much of her time and attention.

Particularly problematic was the treatment by immigration authorities, where there was, as one participant reported “a discord between migration law and every other aspect of the efforts to eradicate violence against women” (Legal practitioner 2). Survivors as well as service providers also raised frustrations with the family law jurisdiction, in particular the prioritization of the perpetrator spending time with children, which appeared to contradict hard-won efforts to separate perpetrators from victims promoted in the family violence civil jurisdiction and/or by child protection authorities.

### Empowerment Through the Legal System

Not all migrant and refugee women in the study felt alienated by the legal system; many women shared stories of empowerment or elements of empowerment amid more challenging experiences. Several participants described perpetrators underestimating their ability to make legal claims because they did not speak English and/or had limited education, and found satisfaction in navigating the legal system:

Afterwards, I gave him the divorce papers. And he couldn't believe it – is this the same woman who came here and couldn't speak English and couldn't drive, who I had to do everything for? I said, ‘I graduated from the university of you’. (Jasmin)

Like Jasmin, successfully participating in the legal system offered the opportunity for some women to express their agency, which may have been stifled during their relationship: “now, in the face of everything that happened, he just can’t believe that someone as stupid as me could drag him around with the police and the courts” (Tabitha). With the help of lawyers, Tabitha collected evidence and presented her case in the Magistrates’ Court for both civil protection and criminal matters, as well as in the family court. She described standing firm against not only the perpetrator in court, but also against family law court officials who encouraged her to settle her case.

As noted above, some women conveyed experiences of empowerment even in circumstances where the legal system proved difficult to navigate or was procedurally unfair, revealing the legal system to be a dynamic and risky site that could result in both experiences of protection and structural discrimination. Li, for example, could not find a legal service to represent her in family court, so she represented herself and won:

I was so helpless. How can I represent myself?… But I decided to fight until the end. So I talked to the legal aid through an interpreter and I asked lots of questions – like what's an affidavit etc – so I did represent myself and got the order. (Li)

It should be noted that two survivors reported that their experience of the legal system positively helped reform the perpetrators’ behavior: “Now, after the court processes, he brings [the kids] toys and talks to them with respect. He's trying, he's trying to improve himself” (Aditi).

Accumulating knowledge of Australian law transformed some migrant and refugee survivors’ views of their entitlement to make legal claims (Merry, 1990). Bilan's partner had threatened she would lose custody if she contacted authorities until she learnt about the law through interactions with family violence support services. Bilan never contacted authorities, but her newfound knowledge changed her former partner's behavior: “I know my legal rights now. So it made me feel more secure…. [And] when I figured out every aspect of legal, he backed back. There wasn’t anything to [manipulate]” (Bilan).

Participants like Bilan who described empowerment often interpreted their positive experiences through the frame of “rights,” underscoring the importance of women feeling justified in making claims of the legal system. Aliyah stated:

Now I feel at ease, and I feel so lucky that I’m in this country because in this country… if there's a woman who's oppressed, she gets all of her rights…. [T]he judge granted me all of my rights and he admitted in front of the judge what he did.

The discourse of rights was mobilized to give expression to feminist notions of “women's rights” for some. Douglas (2012) points out that though the legal system can fail women, contact with the system can still be a context in which women can reframe their experiences through a feminist lens which can be empowering in itself. The experience of gaining or asserting rights through the legal system often informed participants’ attitude toward the state generally: “I feel like the government is on my side because they gave me my rights…The police are on my side, the courts are on my side… I feel like the government supports oppressed women, women in general” (Sandra).

Several service providers described using the language of rights to help support migrant and refugee women in violent relationships and encourage them to engage with the legal system: “I think information about someone's legal rights - it's power” (Legal practitioner 4). One noted however, that her clients rejected the discourse of “rights” when not coupled with access to adequate material/financial support. These clients say: “Why should I work so hard and put myself on Centrelink payment and live with the dole money because I want my rights? Who cares about those rights?” (Caseworker 2). Clearly, abstract notions of legal rights are but just one facet of migrant and refugee women's lives, and such discourse is likely of limited utility when placed in opposition to the fulfilment of basic material needs.

## Discussion and Conclusion

The study indicated that experiences of support provided by the legal system to migrant and refugee women following family violence were inconsistent. Concerningly, many migrant and refugee survivors’ voices were silenced; their claims were not investigated by authorities or heard in court. Treatment often constituted institutional racism, with basic measures of procedural fairness regularly falling short. The research supports policy reforms aimed at making courts inclusive by promoting workforce diversity; providing accessible, translated documents; ensuring adequate time both in court and with legal representatives; and ensuring adequate access to interpreters. Police procedures and training should continue to address the systemic and cultural factors that lead to migrant and refugee survivors being ignored, misunderstood, or misidentified as primary aggressors.

Notwithstanding this, the research also demonstrated that some survivors experienced support and empowerment within the legal system following family violence. There was not a strict binary between individuals who felt empowered or alienated by their interactions with the legal system; various survivors reported ambivalence or experiences of dissonance. Indeed, women's dual experiences of empowerment and alienation confirmed the justice system to be a risky and equivocal environment for survivors, which could offer protection amidst structural discrimination, at times simultaneously.

We found a relationship between survivors’ experiences of the legal system and their conception of the state generally. Women who experienced overarching empowerment within the legal system tended to report feelings of security in Australia and better relationships with the state, whereas survivors largely dissatisfied with the legal system were more likely to describe broader alienation. Indeed, the findings indicate that how the legal system responds to migrant and refugee survivors influences their understanding of their position in relation to the state and the state's law. In their investigation of overpoliced communities in the United States, Prowse et al. (2020) found that people interpreted their interactions with police as an analogy for state treatment, communicating the state's attitudes toward them and their communities. Monica Bell (2017, p. 2054) theorizes that consistent poor treatment by law enforcers can manifest in “legal estrangement,” which “reflects the intuition among many people… that the law operates to exclude them from society.” This concept resonates with discussions of legal alienation and “against the law” narratives within legal consciousness scholarship that focus on disconnection from the law and legal system (Ewick & Silbey, 1998; Halliday, 2019; Hertogh, 2018). Bell's (2017) conception of legal estrangement emphasizes that it is a relational experience, shaped not only by individual encounters but the experiences of the wider community. Exploring how migrant and refugee women's individual experiences of empowerment and/or alienation in the legal system influence collective legal consciousness within communities warrants further inquiry. Nonetheless, it appears likely that there is bidirectional relationship between positive experiences of the legal system and broader social integration.

Our research also noted the importance of the ways in which legal mechanisms and interactions frame particular types of violence experienced by migrant and refugee women. We found a tension between a desire for formal legal recognition of the specific contours of violence that migrant and refugee women face, such as marriage and divorce-related violence, and the danger that migrants and refugees are further racialized through legal frameworks and interactions with the legal system.

For migrant and refugee communities who are already facing societal discrimination or alienation, legal mechanisms which are protective rather than punitive are likely to be most effective. Other research has found that migrant and refugee women are significantly less likely than women in privileged positions to seek legal interventions that punish or arrest their partner (Barrett et al., 2011; Bui, 2003). Our findings indicate that nonpunitive approaches are particularly likely to benefit migrant and refugee women who wish to stay in relationships, as punitive legal responses remain a significant deterrent to their engagement. Insights such as this are likely relevant to all populations, not just refugee and migrant women. How the legal system can best offer protection to migrant and refugee women (and women in general) who wish to maintain their relationship and support their agency requires further exploration.

Women's narratives of empowerment underscore the legal system's potential to promote the well-being of migrant and refugee survivors, though it remains a site of struggle and ambivalence (Marshall & Barclay, 2003). Our findings show that survivors were more satisfied with legal responses by police and courts following family violence where authorities listened to their stories, responded to their claims, and recognized women's ability to identify what was in their own best interests. Such findings unsurprisingly necessitate that procedural justice be afforded to refugee and migrant communities, including provision of adequate interpreter services and accessible legal documents. They also call into question legal approaches that can diminish survivors’ agency (Hourani et al., 2022), such as proarrest policies. The accumulation of accurate legal knowledge was viewed as empowering by many survivors and service providers, helping women navigate the legal system or, in some instances, change perpetrators’ behaviors. Women's reflections highlighted the importance of their conceptions of rights, even in the face of procedural injustices. This finding supports the promotion of efforts to provide meaningful legal education that is tailored to particular populations through community and women's groups or bicultural outreach workers.

Our emphasis on legal education does not suggest, however, that empowerment through the legal system relies or should rely on individual women's knowledge of their rights. Systemic reform efforts must address institutional discrimination evident in this study to support migrant and refugee women's empowerment at a structural level. As well as reforms in police and court approaches, fair treatment by the Australian immigration regime is integral to this effort and necessitates clear pathways of support for all survivors of violence. Furthermore, empowerment through the legal system cannot be separated from reforms in social support and housing that enable migrants and refugees to meet their needs, whether or not they are in a relationship. Survivor-centered legal responses to family violence should recognize the intersecting factors that shape migrant and refugee women's lives and create meaningful space for their agency, allowing them to make claims of the legal system that will be heard.

## Notes

1. In Australia, a person's visa may be canceled for family violence “regardless of whether there is a conviction for an offence or a sentence imposed” (Ministerial Direction No. 90, 2021, para. 8.8.1(1)(a)(iii)).
